# Multilingual Digital Search Behavior Reveals Cultural Disparities in Vitiligo Information Needs in Germany

**DOI:** 10.1111/ijd.70385

**Published:** 2026-03-09

**Authors:** Lilian Rauch, Christine Gasteiger, Markus Böhm, Georg Pliszewski, Tilo Biedermann, Alexander Zink

**Affiliations:** ^1^ Department of Dermatology and Allergy, Biederstein Technical University of Munich Munich Germany; ^2^ Department of Dermatology University Hospital Münster Münster Germany; ^3^ German Vitiligo Association e.V. Adelsdorf Germany

**Keywords:** cultural health disparities, digital epidemiology, Germany, health information needs, multilingual, online search behavior, vitiligo

## Abstract

**Background:**

Vitiligo can cause substantial psychosocial distress, including stigma, depression, and social exclusion. Cultural factors shape disease perception and information needs. Online search queries provide real‐time indicators of unmet public interests. We investigated language‐ and culture‐associated differences in vitiligo‐related online information seeking in Germany to inform culturally competent dermatologic care.

**Methods:**

We conducted a retrospective observational study using anonymized data from the Google Ads Keyword Planner (October 2019–May 2023). Search terms related to vitiligo were analyzed in six languages (German, Turkish, Arabic, English, Russian, Polish), representing the most commonly spoken languages in Germany. Keywords were thematically categorized and comparatively analyzed across languages.

**Results:**

A total of 7.8 million vitiligo‐related search queries were identified, with the majority in German. “General information” was the most frequently searched category, except in Arabic, where “treatment options” ranked highest. Treatment queries in Turkish and Arabic more frequently mentioned alternative therapies and home remedies than specific evidence‐based treatments. Notable cross‐language differences emerged in searches for camouflage, depigmentation, faith‐related coping, and psychosocial burden.

**Conclusions:**

Online search behavior reveals both shared and language‐specific interests about vitiligo. Observed language patterns are hypothesis‐generating and may reflect differences in information access, healthcare navigation, and language barriers rather than inherent cultural preferences. Language‐specific patterns highlight the need for culturally sensitive communication, multilingual patient education, and inclusive care models in dermatology to promote health equity.

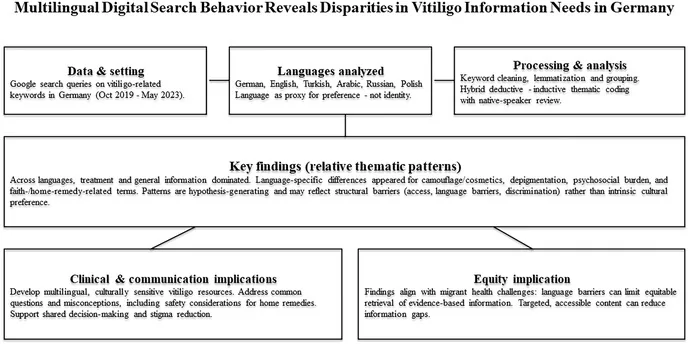

## Plain Language Summary

Many people use the internet to learn about vitiligo, but not everyone searches in the same language or finds the same quality of information. We analyzed Google search terms about vitiligo in Germany across multiple languages to identify differences in what people want to know. While common topics included causes, symptoms, and treatment, the relative emphasis varied by language. This suggests that some communities may face gaps in accessible, trustworthy information. Providing high‐quality vitiligo resources in multiple languages and addressing specific questions of different language groups could support more equitable access to care and patient education.

## Graphical abstract

Multilingual vitiligo‐related Google search queries in Germany (October 2019–May 2023) were extracted and thematically coded across German, English, Turkish, Arabic, Russian, and Polish. Relative patterns highlighted language‐specific emphases in treatment information, camouflage, depigmentation, psychosocial burden, and faith/home‐remedy terms, indicating potential equity‐relevant information gaps due to language barriers.

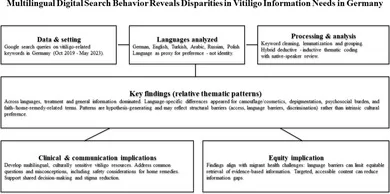

## 1 Introduction

Vitiligo is a chronic, non‐contagious autoimmune disease characterized by the selective destruction of melanocytes, resulting in depigmented skin patches [1, 2]. Globally, the disease affects an estimated 0.7%–1.2% of the population and occurs across all ethnicities, sexes, and age groups [3]. Although not life‐threatening per se, vitiligo can have profound psychosocial consequences. It frequently disrupts self‐image, impairs interpersonal relationships, and contributes to emotional distress, social withdrawal, anxiety, and depression [4, 5, 6, 7]. The visibility of skin changes often renders the condition particularly stigmatizing in societies where uniform skin tone is linked to beauty, health, or social status.

Cultural context significantly shapes how individuals perceive, respond to, and seek care for vitiligo. Existing literature underscores the role of social norms, religious beliefs, and beauty ideals in mediating patient experiences across different communities [8, 9, 10, 11]. In some cultural settings, vitiligo is erroneously associated with contagion or moral impurity, further intensifying stigma and marginalization. These cultural dynamics not only influence coping strategies but also determine whether individuals seek medical help or turn to alternative, cosmetic, or spiritual remedies.

In Germany, approved treatments for vitiligo include topical corticosteroids and calcineurin inhibitors, narrowband ultraviolet B (NB‐UVB) phototherapy, and—in select cases—systemic immunomodulatory agents [1, 12]. As of May 2023, the topical Janus kinase (JAK) inhibitor ruxolitinib has been authorized for non‐segmental vitiligo with facial involvement in individuals aged 12 and older [13]. Meanwhile, several clinical trials investigating oral JAK inhibitors continue to advance the therapeutic landscape [14, 15, 16]. Despite these medical advances, access to specialized dermatologic care remains uneven. Regional disparities and long waiting times—exceeding 8 weeks in some areas—pose significant barriers, particularly for those with limited German proficiency or restricted healthcare literacy [5, 17, 18].

In parallel, the internet has become a primary source of health information. A majority of patients now turn to digital platforms for self‐education, particularly when medical consultations are delayed or inaccessible [19, 20]. In Germany, nearly 75% of adults use the internet for health‐related information, a proportion that rises further among younger and migrant populations [21]. In this context, search engine queries reflect not only biomedical interest but also socio‐cultural interpretations of disease, personal health anxieties, and community‐level knowledge gaps [22, 23, 24].

However, traditional epidemiological data—such as claims databases or clinical registries—rarely account for the diversity of health beliefs or behaviors within multicultural societies. Language, in particular, can serve as an imperfect proxy for linguistic preference and—partly—cultural background, but it does not map one‐to‐one to identity; English‐language searches, for example, may capture diverse groups (e.g., expats, second‐language users, or default device settings) rather than a coherent cultural group.

Analyzing online search queries in users' native languages may therefore reveal latent disparities, including unmet needs, non‐medical coping strategies, and culturally framed understandings of disease [25, 26, 27].

This study aims to systematically analyze language‐specific online search behavior related to vitiligo in Germany, using data from six of the country's most spoken languages. By identifying thematic patterns and culturally influenced interests, the study seeks to shed light into more inclusive public health communication, culturally competent dermatologic care, and equitable resource allocation for chronic skin conditions.

## 2 Methods

### 2.1 Study Design and Data Source

This retrospective, observational study analyzed language‐specific online search behavior related to vitiligo in Germany. The study employed the Google Ads Keyword Planner as its primary data source—a tool widely used in health informatics and digital epidemiology research for extracting aggregated search frequency data [28]. The tool compiles monthly search volume estimates based on user behavior on Google Search, which accounts for more than 95% of the search engine market share in Germany during the observation period [29]. The data reflect anonymized, population‐level search activity, free from personally identifiable information. As such, ethical approval was not required.

This approach aligns with prior dermatologic research leveraging web‐based analytics to understand public interest, knowledge gaps, and unmet needs in skin diseases [30, 31, 32, 33, 34, 35]. By capturing spontaneous, real‐world queries entered by users in their native language, this method offers unique access to socio‐cultural dimensions of illness perception that traditional clinical or survey data may not capture.

### 2.2 Language Selection and Search Strategy

To ensure representation of Germany's multicultural population, we selected the six most widely spoken languages in the country: German, Turkish, Arabic, English, Russian, and Polish [36]. These languages collectively represent the primary linguistic communities in Germany beyond native German speakers and are associated with both long‐established migrant groups and more recent arrivals.

For German, two seed terms were used to initiate the keyword query process within the Google Ads Keyword Planner: the clinical term “vitiligo” and the colloquial synonym “white spot disease” (Weißfleckenkrankheit). For the other five languages, only the clinical term “vitiligo” was used as the initial seed term. These entry terms were selected based on their frequency in lay communication and their capacity to return a broad yet disease‐specific set of associated queries. Search results included directly related terms and semantically linked queries as determined by Google's proprietary algorithms.

The observation period spanned from October 2019 to May 2023. This window was chosen to balance sufficient longitudinal data with clinical relevance. May 2023 marked the European approval of ruxolitinib cream for non‐segmental facial vitiligo—a major therapeutic milestone that may have influenced digital information‐seeking behavior [13]. Limiting the analysis to pre‐approval data ensured consistency in the treatment landscape throughout the study period.

### 2.3 Keyword Processing and Thematic Categorization

The raw output for each language was cleaned to remove duplicates, low‐volume entries, and irrelevant or overly generic terms. Queries containing nonspecific symptom descriptions (e.g., “white dots”) were excluded unless clearly context‐linked to vitiligo. The remaining terms were subjected to a multi‐step translation and validation process. First, non‐German terms were translated into German using Google Translate and Linguee. Second, the semantic accuracy and cultural relevance of translations were reviewed by native‐speaking members of the research team. This step was crucial for avoiding misclassification due to idiomatic or culturally specific language.

We then performed thematic categorization using a hybrid coding approach. This involved:

- Deductive categorization, guided by existing literature on vitiligo's clinical, psychosocial, and cultural dimensions [4, 5, 37, 38, 39],
- Inductive coding, allowing new, unanticipated themes to emerge from the language‐specific data sets.

The initial translation and thematic coding of keywords were performed independently by bilingual coders with cultural fluency in the respective language. Coding consistency was enhanced by iterative team discussions, during which ambiguities were clarified and categories refined. Discrepancies were resolved through consensus. This multi‐layered coding process ensured both methodological rigor and cultural sensitivity.

The final thematic framework comprised seven top‐level categories:

1. General Information—Broad queries including definitions, causes, skin color descriptors, and age‐specific concerns (e.g., “vitiligo in babies”).
2. Symptoms—Terms referring to depigmentation patterns, discoloration, and associated physical signs.
3. Affected Body Areas—Including face, arms and hands, hair, genital area, upper body, legs and feet, and subtype‐specific terms like “acrofacial” or “mucosal vitiligo.”
4. Treatment Options—Subdivided into:
  - General therapeutic information
  - Evidence‐based medical therapies (e.g., corticosteroids, UV therapy)
  - Light‐based therapies (e.g., laser, NB‐UVB)
  - Alternative treatments and home remedies (e.g., dietary supplements, herbal products, vinegar)
  - Camouflage and depigmentation (e.g., makeup, permanent tattooing, skin bleaching)
5. Psychosocial Aspects—Including terms related to mental health, social stigma, emotional burden, and quality of life.
6. Experts and Specialized Care—Queries seeking dermatologists, clinics, or treatment centers for vitiligo.
7. Miscellaneous—Including celebrity references, pets with vitiligo, differential diagnoses (e.g., pityriasis versicolor), and pandemic‐related searches (e.g., “vitiligo after COVID”).

### 2.4 Data Analysis

Search volumes were aggregated by language and thematic category. Monthly search volumes were summed across the study period to obtain cumulative frequencies. Descriptive statistics were calculated using Python. The distribution of themes was compared across languages to identify both common and divergent patterns in search behavior. Particular attention was given to themes of cultural relevance such as alternative medicine, religious healing, and psychosocial burden.

To maintain interpretive fidelity, qualitative comparisons were used to complement numerical differences. Keyword examples were reviewed contextually to assess how health beliefs, cultural norms, and emotional experiences might shape search behavior in each linguistic community.

## 3 Results

The total search volume was highest in German (3,162,260), followed by English (2,428,440), Turkish (1,667,580), Arabic (367,460), Russian (126,810), and Polish (11,530). These absolute volumes reflect overall search activity and are not normalized to the size, internet use, or prevalence of each language community in Germany; therefore, cross‐language comparisons should primarily rely on relative distributions across thematic categories.

The most prevalent search category across all languages was ‘General information’, except for Arabic, where ‘Treatment options’ was the most popular search term. A particularly salient feature was the high frequency of keywords related to ‘babies’ and ‘children’ within general information searches. A comparative analysis of keywords related to skin tone in German and Turkish revealed a greater prevalence of terms associated with ‘light skin’. Conversely, English, Arabic and Russian exhibited a higher frequency of keywords related to ‘lighter’ and ‘darker skin’.

Search terms assigned to the category ‘treatment options’ constituted the second most frequently requested category, with the exception of Arabic, where the category was the most frequently requested. This indicates that this category is distinguished by significant linguistic differences. In Turkish and Arabic, the most prevalent subcategory of queries pertained to alternative medical treatments and home remedies, such as red clay, vinegar, and food. Conversely, in German, English, and Russian, the most frequent searches were related to commercial products and dietary supplements. In this study, “homeopathy” and “acupuncture” emerged as the most commonly searched terms in German and English. Conversely, the terms “cupping” and “acupuncture” were more frequently entered in Arabic and Turkish. In addition, searches were conducted in both German and Russian for traditional Chinese medicine. A considerable number of search terms in Turkish, Russian, and Arabic focused on treatment through faith, Islam, and the Koran. A common feature across all languages was that terms relating to evidence‐based therapies were searched for less frequently than alternative medicine therapies. A significant number of search terms relating to camouflage or depigmentation were identified in English, Turkish, and Arabic. Conversely, no such terms were identified in German, Russian, and Polish.

The category designated ‘Miscellaneous’ was the third most frequently searched category across all languages. There were discernible discrepancies in the content. In the German corpus, the search term ‘dogs’ was entered with a high frequency. Frequent searches linking vitiligo to COVID‐19 infection were observed in the German and Turkish datasets. A substantial number of celebrity‐related queries were observed in both the English and Russian datasets, with distinct individuals predominant in each language group. Furthermore, the ‘Miscellaneous’ category was assigned differential diagnoses. The most prevalent search term in German was for pityriasis versicolor, a skin fungus, while in English, the most common search term was leukoderma. In Arabic, the most prevalent search terms were leprosy and albinism. Religion‐related keywords were identified in the Arabic, Turkish, Russian, and English datasets.

The terms from the category ‘symptoms’ were the fourth most frequently searched, followed by ‘affected body areas’ in fifth place. In all languages, the majority of searches related to the genitoanal area, face, hair, and arms were observed, with the exception of Arabic, where there were hardly any searches for the genitoanal area.

The sixth most prevalent category was ‘psychosocial aspects’. In the Polish dataset, the terms ‘vitiligo’ and ‘stress’ appeared with notable frequency.

The analysis revealed that terms from the ‘experts’ category were the least frequently searched for.

## 4 Discussion

This study provides the first systematic analysis of language‐specific online search behavior relating to vitiligo in Germany. By leveraging data across six of the most commonly spoken languages, we identified both universal and culturally specific patterns in health‐related information needs. These insights extend beyond raw search volumes—they reflect sociocultural understandings of disease, differing concepts of causality and healing, and potential structural gaps in care provision [4, 37, 38, 39]. In doing so, the study contributes meaningfully to the growing fields of digital epidemiology, health disparities research, and culturally competent dermatologic care [40].

A key finding is the consistently high interest in treatment‐related information across all language groups, underscoring a persistent need for effective therapies and accessible guidance [5, 8, 41]. However, the content of treatment‐related queries differed by language. Turkish‐ and Arabic‐language queries more often included terms related to alternative remedies, home treatments, and faith‐based coping [42, 43, 44, 45, 46, 47]. This pattern should not be interpreted as an inherent cultural preference; it may also reflect structural barriers such as limited access to dermatologic care, language proficiency, discrimination, or prior negative experiences with the healthcare system. The English‐language dataset additionally showed interest in camouflage and depigmentation, which may relate to diverse user groups and different stigma experiences [3, 37, 48, 49]. These language‐associated preferences point to broader themes of trust, representation, and accessibility within dermatologic care.

Our findings align with existing literature on the psychosocial burden of vitiligo, which includes diminished quality of life, increased anxiety, and social withdrawal—particularly among people of color and marginalized groups [4, 50]. In Germany, where regional and structural disparities in dermatologic access remain prevalent [17, 18], language and culture may compound these inequities. Taken together, the language‐specific query patterns are consistent with migrant health challenges, suggesting that language barriers and limited availability of culturally appropriate information may hinder equitable retrieval of evidence‐based vitiligo resources. This study shows that even within a high‐income country, structural exclusion can persist through less visible channels such as information access, language barriers, and cultural incongruence in health communication.

From a methodological standpoint, the use of Google search data has inherent limitations. Google's proprietary algorithms may influence which terms are suggested or prioritized, potentially skewing the data. Additionally, search queries do not reveal demographic specifics such as age, gender, or confirmed diagnosis. Absolute search volumes were not normalized to the size or internet use of each language community, and language is an imperfect proxy for cultural identity—particularly for English‐language searches. Because the observation period overlapped with the COVID‐19 pandemic (October 2019–May 2023), search volumes and content may have been influenced by pandemic‐related attention; while COVID‐related keywords appeared in several corpora (notably German and Turkish), we did not perform formal pandemic vs. post‐pandemic subgroup analyses.

These findings offer concrete guidance for clinical dermatology in multicultural settings. The high frequency of non‐German queries—especially those referencing alternative remedies, faith‐related coping, and camouflage—suggests that existing patient education may not fully match the questions and contexts of diverse communities. Dermatologists and educators should co‐develop multilingual, culturally attuned resources that promote evidence‐based options while respectfully addressing traditional practices, beliefs, and safety considerations. Indeed, culturally competent counseling, multilingual patient portals, and targeted community outreach could reduce stigma, enhance adherence, and foster trust [51, 52]. This is also a cornerstone in the shared decision‐making process that is crucial for the therapeutic management of vitiligo [16].

Beyond individual practice, the study also has implications for dermatologic health policy and planning. Digital search data can complement traditional epidemiological sources by capturing unmet needs in real time across population segments. In future, health systems might integrate digital behavioral analytics to identify regional or cultural care gaps and develop responsive, equity‐oriented interventions. For example, localized campaigns in Arabic‐ or Turkish‐speaking communities could address misinformation, increase awareness of new therapies such as JAK inhibitors, and provide culturally relevant support for psychosocial burden.

Ultimately, this study underscores that while vitiligo may be uniform in clinical definition, its lived experience is shaped by culture, language, belief systems, and social context. Advancing a fair, inclusive, and needs‐responsive dermatology requires moving beyond a one‐size‐fits‐all model. It requires deliberate integration of cultural humility, linguistic diversity, and digital literacy into care pathways. By doing so, we move closer to a dermatologic practice that is not only scientifically sound but also socially responsive and globally relevant (Figures 1, 2, 3, 4).

**FIGURE 1:**
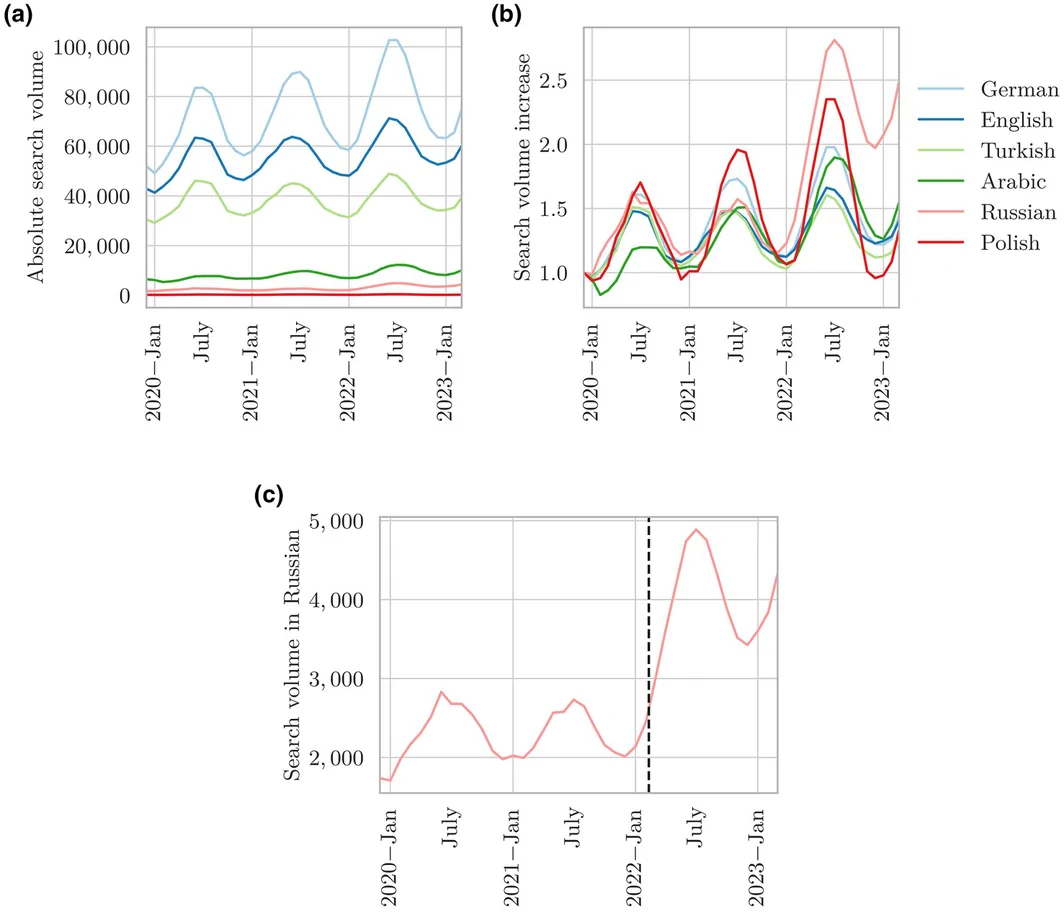
Five‐month centered rolling average of monthly search volumes in Germany for each language. Left: (a) Absolute search volume. Right: (b) Search volume indexed to December 2019. (c) Five‐month centered rolling average of monthly search volume for Russian, showing a marked surge in spring 2022 (vertical dashed line indicates 24 February 2022, the onset of the Russian invasion of Ukraine), exceeding normal seasonal variation.

**FIGURE 2:**
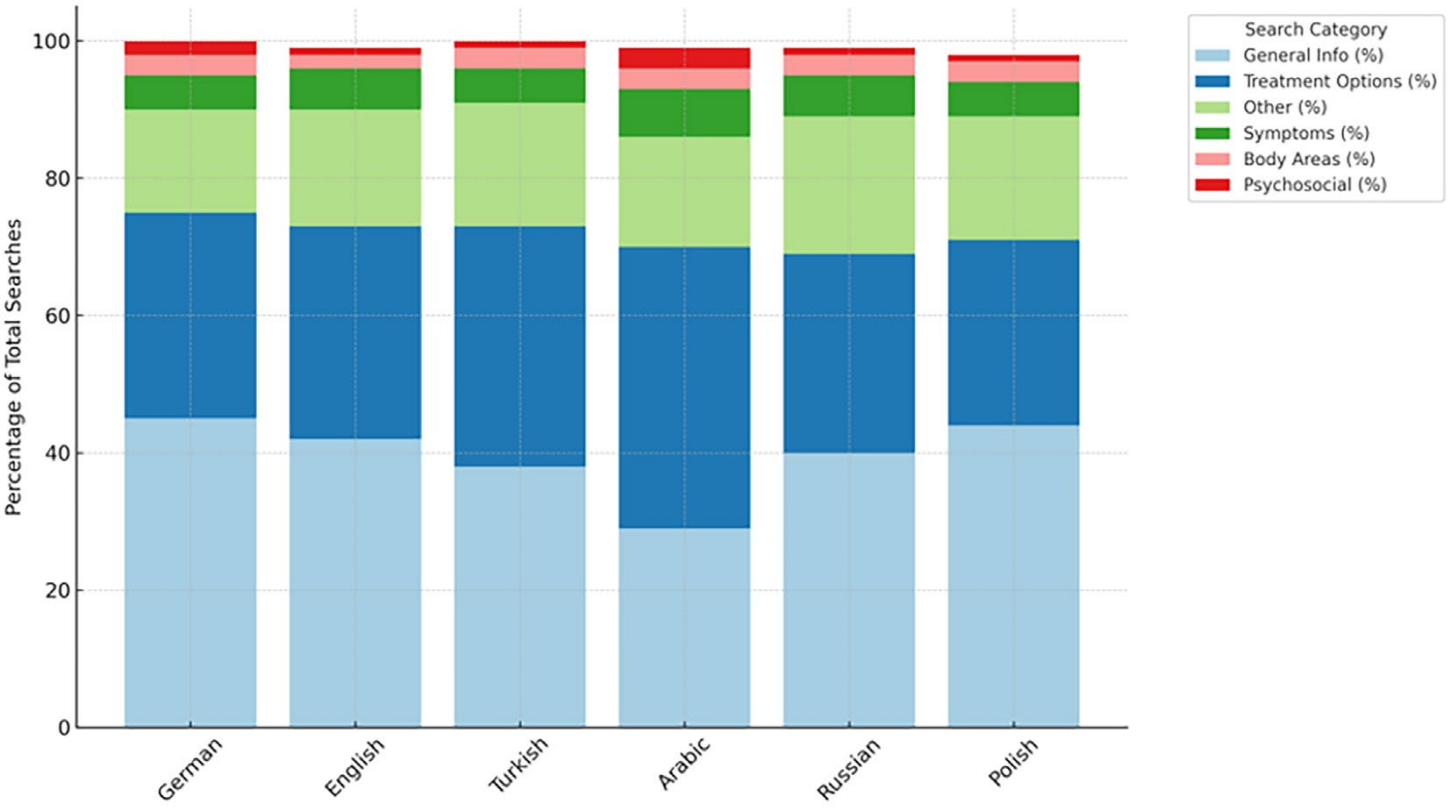
Distribution of vitiligo‐related search categories by language.

**FIGURE 3:**
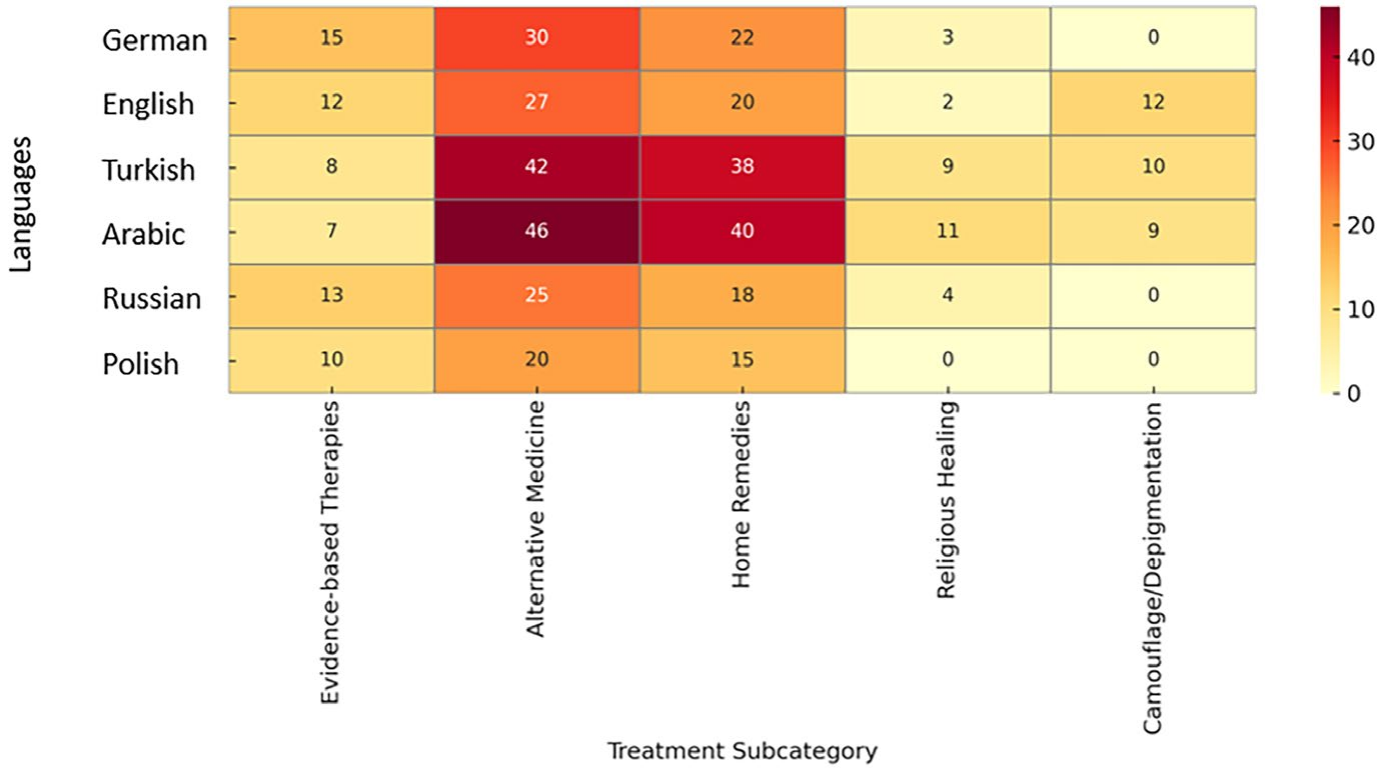
Cultural patterns in treatment‐related searches for vitiligo.

**FIGURE 4:**
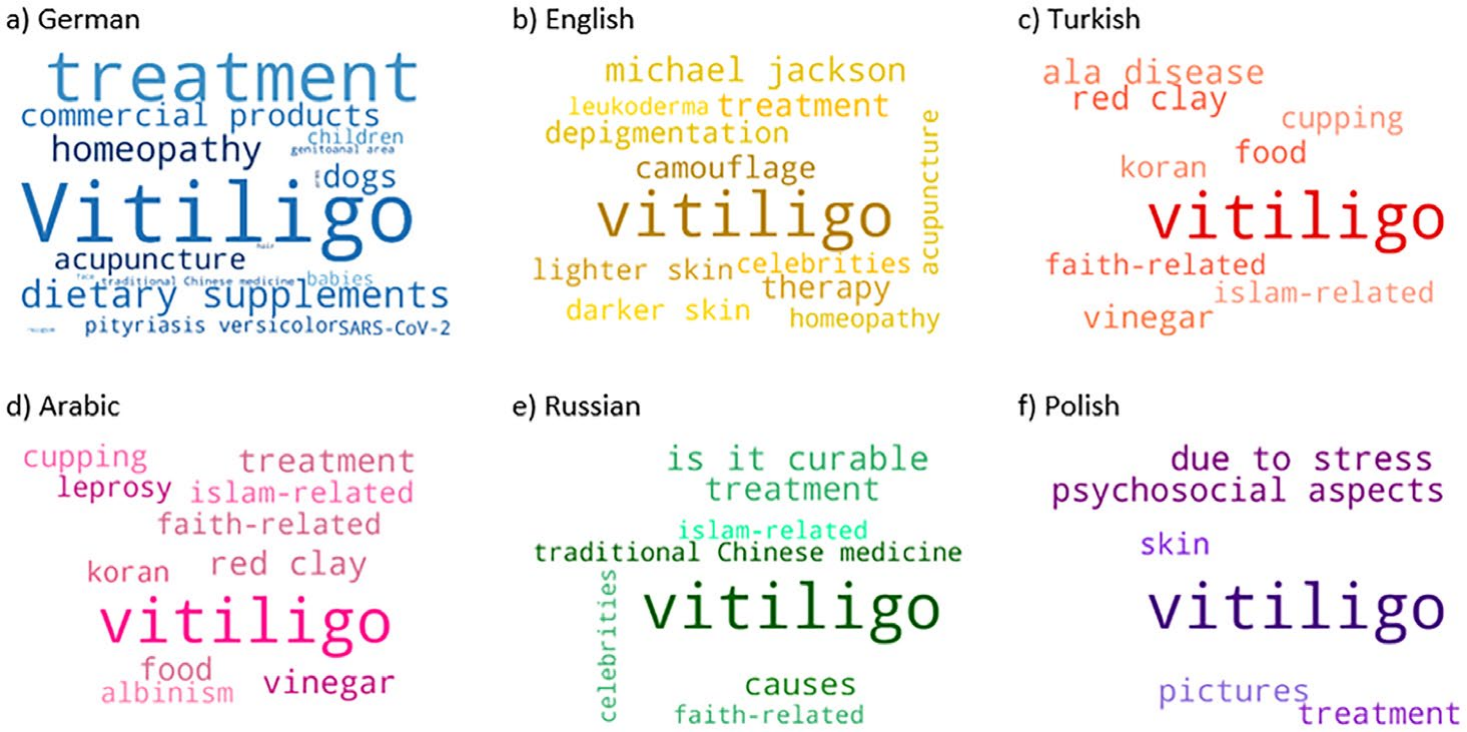
Language‐specific vitiligo‐related search terms in Germany.

## 5 Conclusion

This study offers the first systematic, multilingual analysis of online search behavior related to vitiligo in Germany, revealing substantial variation in culturally influenced information needs across linguistic groups. While a strong interest in therapeutic options was universal, the prominence of queries related to alternative medicine, religious healing, and cosmetic concerns in specific populations points to deeper cultural perceptions of disease and potential mismatches with conventional healthcare delivery.

These findings underscore the potential of digital search data as a sensitive and scalable indicator of health disparities—particularly among linguistically or culturally diverse populations. They also make a compelling case for the integration of culturally sensitive communication, inclusive patient education strategies, and multilingual resources into dermatologic practice.

As vitiligo care enters a new era with novel treatments like JAK inhibitors, these insights can inform more equitable access, improved patient engagement, and greater cultural resonance in therapeutic conversations. Future research should extend this approach through international comparisons, qualitative validation, and integration with clinical outcomes to inform the development of a more inclusive, data‐driven, and human‐centered dermatology.

## Funding

This work was supported by an unrestricted research grant from Incyte Biosciences Germany GmbH. The funding source had no role in the study design, data collection, analysis, or manuscript preparation.

## Conflicts of Interest

The authors declare no conflicts of interest.

## Data Availability

The data that support the findings of this study are available from Google Ads Keyword Planner (Google LLC). Restrictions apply to the availability of these data, which were used under license for this study. Data are available from the author(s) with the permission of Google Ads Keyword Planner (Google LLC).
